# Diagnostic performance of oral swabs for non-sputum based TB diagnosis in a TB/HIV endemic setting

**DOI:** 10.1371/journal.pone.0262123

**Published:** 2022-01-13

**Authors:** Sylvia M. LaCourse, Evans Seko, Rachel Wood, Wilfred Bundi, Gregory S. Ouma, Janet Agaya, Barbra A. Richardson, Grace John-Stewart, Steve Wandiga, Gerard A. Cangelosi

**Affiliations:** 1 Division of Allergy and Infectious Diseases, Department of Medicine, University of Washington, Seattle, WA, United States of America; 2 Department of Global Health, University of Washington, Seattle, WA, United States of America; 3 Kenya Medical Research Institute (KEMRI), Kisumu, Kenya; 4 Department of Environmental & Occupational Health, University of Washington, Seattle, WA, United States of America; 5 Department of Biostatistics, University of Washington, Seattle, WA, United States of America; 6 Department of Epidemiology, University of Washington, Seattle, WA, United States of America; 7 Department Pediatrics, University of Washington, Seattle, WA, United States of America; Indian Institute of Technology Delhi, INDIA

## Abstract

**Objective:**

We evaluated diagnostic performance of oral swab analysis (OSA) for tuberculosis (TB) in a high HIV/TB burden setting in Kenya.

**Methods:**

In this cross-sectional study, buccal swabs and sputum were collected from 100 participants with suspected TB in outpatient clinics in Kenya at enrollment and subsequent morning visits. Buccal swabs underwent IS6110-targeted qPCR analysis. Sputum was evaluated by Xpert MTB/RIF (Xpert) and culture. Diagnostic performance of OSA for TB diagnosis was evaluated relative to a combined reference of sputum Xpert and culture.

**Results:**

Among 100 participants, 54% were living with HIV (PLHIV). Twenty percent (20/100) of participants had confirmed TB (19/20 [95%] culture-positive, 17/20 [85%] Xpert-positive). Overall buccal swab sensitivity was 65.0% (95% CI 40.8–84.6%) vs. sputum Xpert/culture and 76.5% (95% CI 50.1–93.2%) vs. sputum Xpert alone. Specificity was 81.3% (95% CI 71.0–89.1%) and 81.9% (95% CI 72.0–89.5%) compared to sputum Xpert/culture and Xpert alone, respectively. Sensitivity among PLHIV (n = 54) with suspected TB was 83.3% (95% CI 35.9–99.6%) vs. sputum Xpert/culture and 100% (95% CI 47.8–100.0%) vs. sputum Xpert alone. Among participants with TB, mean OSA threshold quantitation cycle (Cq) value was lower (stronger signal) at subsequent morning compared to enrolment visit (33.4 SD ± 3.7 vs. 35.2 SD ± 2.9, p = 0.009).

**Conclusions:**

In this pilot study, results confirm *M*. *tuberculosis* DNA is detectable in oral swabs including among PLHIV with fair diagnostic performance. Further work is needed to optimize OSA and evaluate its utility in diverse settings.

## Introduction

Tuberculosis (TB) is a leading infectious cause of mortality globally, particularly among people living with HIV (PLHIV) [1]. Despite advances in TB diagnostics, such as Xpert MTB/RIF (Xpert) [2] and Xpert-Ultra [3], sample collection remains challenging especially in those unable to produce sputum [4]. *M*. *tuberculosis*, like most bacteria, has evolved mechanisms to adhere to cell surfaces. Our previous oral swab analysis (OSA) studies confirmed bacilli accumulate on oral epithelium of pulmonary TB patients [5, 6]. In a pilot study that sampled buccal surfaces (3 swabs per patient), OSA identified 18/20 (90%) of sputum Xpert-positive individuals without HIV in South Africa, with 100% specificity among healthy negative controls (20/20) in Seattle, Washington [5]. Among children with TB in South Africa, OSA using buccal swabs matched or exceeded diagnostic yield of induced sputum [7].

OSA provides an easy-to-use, non-invasive means for sample collection, and unlike sputum does not produce potentially infectious aerosols. A low-cost, non-sputum based means to diagnosis TB could be a “game-changer” with particular impact for PLHIV [8].

We evaluated diagnostic performance of OSA using buccal swabs among symptomatic individuals with suspected TB in western Kenya, an area of high HIV/TB burden. We hypothesized *M*. *tuberculosis* would be detectable using OSA including among PLHIV.

## Methods and analysis

### Study design and participants

We performed a cross-sectional observational study evaluating OSA diagnostic performance to detect pulmonary TB at outpatient clinics among individuals with suspected TB (cough for 2 weeks plus weight loss, fever, and/or night sweats) evaluated by buccal swabs and sputum for Xpert/culture. This definition of suspected TB has been used previously by other diagnostic performance evaluations jointly performed by the KEMRI/CDC in Kenya [9, 10]. Individuals ≥13 years of age on anti-TB treatment for ≤7 days were eligible. Participants were enrolled consecutively at KEMRI/CDC Clinical Research Center-affiliated clinics in Kisumu, Kenya, an area of high HIV/TB burden [11, 12].

### Procedures

On enrollment, standardized questionnaires regarding socio-demographic, HIV, TB history and symptom screen [13] were administered by study staff. Participants with suspected TB were seen at enrollment and a consecutive morning visit. At both visits, two buccal swabs (left and right buccal surface) and a control swab (exposed to air only) were collected as previously described [5]. Sputum was then collected for AFB smear, Xpert MTB/RIF (Xpert), and culture (MGIT, Manual Mycobacterial Growth System) performed at ISO 15189-accredited KEMRI/CDC TB Laboratory, Kisumu, Kenya.

Buccal swab collection and processing has been previously described [5, 6]. Briefly, OmniSwabs (Whatman, Maidstone, UK) were used with the head of the swab immediately ejected into a collection tube containing sterile lysis buffer. Samples were stored in a cooler box at 2–8°C at the clinic site and transported to KEMRI/CDC TB Laboratory within 8 hours of collection then stored at -80°C.

Cryopreserved swabs underwent DNA extraction and qPCR analysis targeting IS6110 insertion sequence unique to *M*. *tuberculosis* complex at University of Washington, Seattle, Washington. A predetermined threshold quantitation cycle (Cq) ≤38 was considered positive (lower Cq indicating higher copies and stronger/more positive signal indicating higher mycobacterial burden) based on previous work [5]. The laboratory team performing OSA was blinded to Xpert and/or culture results; OSA results were not available to the clinical team at time of Xpert and/or culture evaluation.

### Outcomes

OSA performance was compared to combined reference standard of sputum Xpert (WHO-recommended first line TB diagnostic) and culture (‘gold standard’ reference). Participants with at least one sputum Xpert or culture positive for *M*. *tuberculosis* were considered to have TB; those with negative Xpert and culture were considered TB negative. OSA positivity was defined as one or more buccal swabs positive for *M*. *tuberculosis*.

### Statistical analysis

Participant characteristics were summarized by frequency and proportion for categorical variables, and by median and interquartile range (IQR) for continuous variables. Generalized linear models with a log link and Poisson family were used to estimate relative risk ratios (RR) to assess differences in baseline characteristics by TB status. We estimated sensitivity and specificity of buccal swab OSA to detect TB compared to reference standard of sputum Xpert and culture using 95% confidence intervals (CI) assuming a binomial distribution. Additional analyses were performed comparing buccal swab OSA to Xpert alone (as it is the WHO-recommended first line TB diagnostic). Secondary analyses included OSA performance stratified by TB/HIV status, visit, and sputum Xpert signal. Mean Cq of OSA buccal swabs were assessed by TB/HIV status and visit and compared using t-tests.

### Ethical considerations

Written informed consent was obtained from all participants and assent and parental permission for participants <18 years. Study procedures were approved by KEMRI Scientific and Ethics Review Unit and University of Washington Institutional Review Board.

## Results

### Participant characteristics

**Fig 1:**
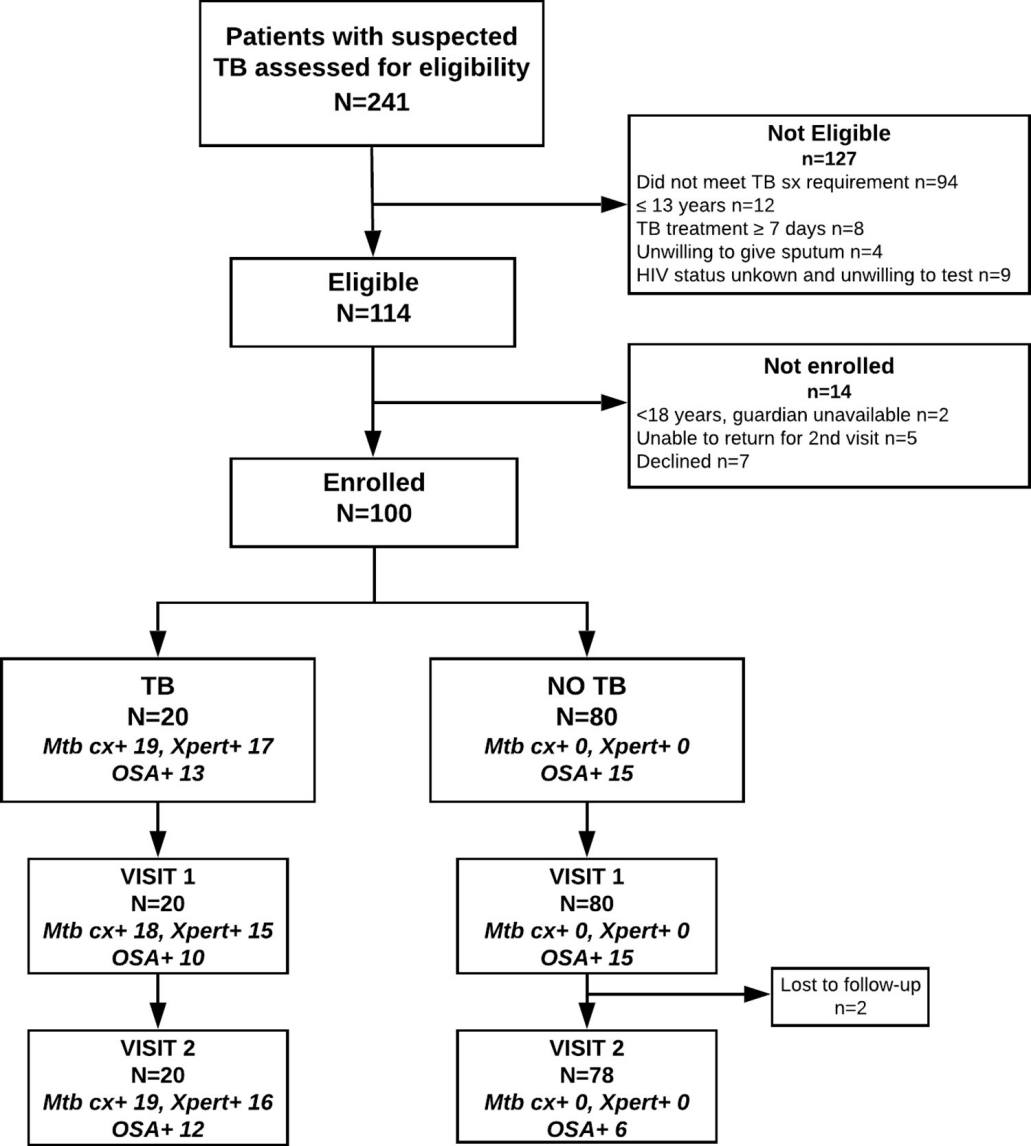
Study flow of participants evaluated for oral swab analysis (OSA) for TB diagnosis in western Kenya. Visit 1 = enrollment, Visit 2 = subsequent morning. TB—tuberculosis, Mtb—*Mycobacterium tuberculosis*, Cx+—culture-positive.

Between November 2016 and March 2017, 100 participants with suspected TB were enrolled (**Fig 1**). Participant characteristics are shown in **Table 1.** Overall, 48 (48%) participants were female and median age was 38 years (IQR 30–44). Twenty (20%) participants reported previous TB. Fifty-four (54%) were PLHIV, including 47 (87%) on antiretroviral therapy (ART), 10 (18.5%) received isoniazid preventative therapy (IPT), and 47 (87%) were taking co-trimoxazole enrollment. All 100 participants reported cough, 85 (85%) fever, 82 (82%) night sweats, 21 (21%) weight loss, and 27 (27%) hemoptysis. Participants with TB (confirmed by culture or Xpert) were younger than participants without TB. Participants with HIV found to have confirmed TB were less likely to be on ART or to have ever received isoniazid preventive therapy (IPT). In this study of symptomatic participants with suspected TB, the proportion of participants with specific TB symptoms were similar between participants with confirmed TB and no TB.

**Table 1:** Characteristics of participants suspected of TB evaluated for oral swab analysis (OSA) using buccal swabs for TB diagnosis in western Kenya.

	Total^a^	TB^b^	No TB^c^	RR^d^ (95% CI)	p
	N = 100	N = 20	N = 80		
	n or median	n or median	n or median		
	(% or IQR)	(% or IQR)	(% or IQR)		
**Sociodemographic**					
Female^e^	48 (48.0)	4 (20.0)	44 (55.0)	0.27 (0.10–0.76)	0.13
Age (years)	38 (30–44)	32 (28–37)	39 (30–47)	0.96 (0.93–0.99)	0.009
Smoker (any)	6 (6.0)	1 (5.0)	5 (6.3)	0.82 (0.13–5.21)	0.84
Etoh (any)	21 (21.0)	7 (35.0)	14 (17.5)	2.03 (0.92–4.45)	0.08
**HIV**	54 (54.0)	6 (30.0)	48 (60.0)	0.37 (0.15–0.88)	0.03
Current ARVs	47 (87.0)	3 (50.0)	44 (91.7)	0.15 (0.03–0.61)	0.008
IPT (ever)	10 (18.5)	0 (0)	10 (20.8)	--	<0.001
Co-trimoxazole	47 (87.0)	43 (89.6)	4 (66.7)	0.30 (0.07–1.35)	0.11
**TB symptoms**					
Cough	100 (100)	20 (100)	80 (100)	--	--
Weight loss	21 (21.0)	3 (15.0)	18 (22.5)	0.66 (0.21–2.06)	0.48
Fever	85 (85.0)	18 (90.0)	67 (83.8)	1.59 (0.41–6.19)	0.51
Night sweats	82 (82.0)	18 (90.0)	64 (80.0)	1.98 (0.50–7.81)	0.33
Breathing difficulty	59 (59.0)	14 (70.0)	45 (56.3)	1.62 (0.68–3.89)	0.28
Hemoptysis	27 (27.0)	6 (30.0)	21 (26.3)	1.16 (0.49–2.72)	0.74
Lymphadenopathy	0 (0)	0 (0)	0 (0)	--	--
History of TB	20 (20.0)	3 (15.0)	17 (21.3)	0.71 (0.22–2.19)	0.55

Abbreviations: TB: tuberculosis; IPT: isoniazid preventive therapy.

^a^ Participants suspected of TB with cough ≥2 weeks as well as at least one additional symptom (fever, weight loss, night sweats, or hemoptysis).

^b^ Either sputum culture or Xpert positive for *M*. *tuberculosis*.

^c^ Negative sputum culture and Xpert for *M*. *tuberculosis*.

^d^ Relative risk (RR) estimated using a generalized linear model (GLM) with log link and Poisson family, reference No TB.

^e^ includes 3 pregnant women, 1 postpartum woman (delivery in past 12 months) all no TB.

### Sputum and OSA sample results

Among 100 participants with suspected TB, 20 (20/100, 20%) were diagnosed with TB by sputum Xpert and/or culture (**Fig 1**). Thirteen percent of participant had any smear positive, 19% any *M*. *tuberculosis* culture positive, and 17% any Xpert positive. Twenty-eight percent of participants had any buccal swabs positive (**S1 Table**). All air control swabs tested were negative for *M*. *tuberculosis*.

### OSA diagnostic performance

Among 100 participants with suspected TB, buccal swab sensitivity was 65.0% (95% CI 40.8–84.6%) vs. sputum Xpert/culture and 76.5% (95% CI 50.1–93.2%) vs. sputum Xpert alone (**Table 2**). Specificity was 81.3% (95% CI 71.0–89.1%) compared to sputum Xpert/culture and 81.9% (95% CI 72.0–89.5%) compared to sputum Xpert alone. Among 54 PLHIV, buccal swab sensitivity was 83.3% (95% CI 35.9–99.6%) vs. sputum Xpert/culture and 100% (95% CI 47.8–100.0) vs. sputum Xpert alone with specificity of 77.1% (95% CI 62.7–88.0%) and 88.2% (95% CI 72.5–96.7%), respectively (**Table 3**).

**Table 2:** OSA diagnostic performance for TB overall and by visit.

	***OSA vs sputum Xpert and/or culture***		
	**Sensitivity (%)**	**Specificity (%)**	**AUC**
**Suspected TB** ^**a**^	13/20 (65.0)	65/80 (81.3)	.731
Visit 1	10/19 (52.6)	70/81 (86.4)	.695
Visit 2	12/20 (60.0)	72/78 (92.3)	.762
	***OSA vs sputum Xpert alone***		
	**Sensitivity (%)**	**Specificity (%)**	**AUC**
**Suspected TB** ^**a**^	13/17 (76.5)	68/83 (81.9)	.792
Visit 1	6/15 (60.0)	73/85 (85.9)	.729
Visit 2	12/16 (75.0)	76/82 (92.7)	.838
	***OSA vs sputum culture alone***		
	**Sensitivity (%)**	**Specificity (%)**	**AUC**
**Suspected TB**^**a**^	12/19 (63.2)	65/81 (80.2)	.717
Visit 1	10/18 (55.6)	71/82 (86.6)	.711
Visit 2	11/19 (57.9)	72/79 (91.9)	.745

AUC—Area under the receiver operating characteristic curve

^a^ 4 buccal swabs collected at 2 visits compared to Xpert and/or culture on 2 sputums

**Table 3:** OSA diagnostic performance by HIV status.

	***OSA vs sputum Xpert and/or Culture***		
	**Sensitivity (%)**	**Specificity (%)**	**AUC**
**Suspected TB** ^**a**^			
HIV+	5/6 (83.3)	37/48 (77.1)	.802
HIV-	8/14 (57.1)	28/32 (87.5)	.723
	***OSA vs sputum Xpert alone***		
	**Sensitivity (%)**	**Specificity (%)**	**AUC**
**Suspected TB** ^**a**^			
HIV+	5/5 (100)	38/49 (77.6)	.888
HIV-	8/12 (66.7)	30/34 (88.2)	.775
	***OSA vs sputum culture alone***		
	**Sensitivity (%)**	**Specificity (%)**	**AUC**
**Suspected TB**^**a**^			
HIV+	5/6 (83.3)	37/48 (77.1)	.802
HIV-	7/13 (53.8)	29/33 (84.8)	.693

AUC—Area under the receiver operating characteristic curve

^a^ 4 buccal swabs collected at 2 visits compared to Xpert and/or culture on 2 sputums

### OSA and Xpert signals

Buccal swabs were positive in 63% to 69% of samples corresponding with samples of very low to medium sputum Xpert signals, and 100% in sputum with high Xpert signals (**S2 Table)**. In general, OSA signals appeared stronger (lower Cq) with corresponding sputums with stronger Xpert signals (**S3 Table**).

### OSA signals by TB, HIV status, and visit

OSA signal was stronger (lower Cq) among individuals with TB vs. without TB at both enrolment (TB: 35.4 SD ± 3.5 vs. No TB: 38.4 ± 3.5, p = 0.03), and subsequent morning visits (TB: 33.2 SD ± 3.6 vs. No TB: 39.2 ± 2.9, p<0.0001) and among participants with TB at the subsequent morning vs. enrolment visit (33.4 SD ± 3.7 vs. 35.2 SD ± 2.8, p = 0.009) (**S4 Table**). OSA signal appeared similar between PLHIV and HIV-negative participants.

## Discussion

In this study, OSA using buccal swabs was able to detect *M*. *tuberculosis* using a non-sputum means of sampling both in people living with and without HIV in a high TB burden setting. There was a trend of improved performance corresponding with stronger sputum Xpert signals and in subsequent early morning collected samples, with similar performance regardless of HIV status.

This pilot study contributes to the growing literature on the use of OSA for TB detection, with a particular focus on a high HIV/TB burden setting. Subsequent to this study, OSA evaluations in South Africa found OSA using tongue swabs showed significantly stronger TB signals (lower quantitative Cq) than buccal swabs concurrently sampled from the same participants [6]. In this South African study, OSA sampling of the tongue dorsa (2 swabs per patient) had 93% sensitivity and 92% specificity relative to sputum Xpert testing [6]. Different swab types were also evaluated and found that PurFlock Ultra swabs (Puritan, Guilford, USA) had a modest improvement in signal vs. OmniSwabs which were used in this Kenyan evaluation [6]. Additional evaluation of a variety of swab types in a Ugandan study revealed that Copan FLOQSwabs collected 2-fold more bacterial biomass than PurFlock Ultra swabs and confirmed the previous findings of improved sensitivity of tongue swabs for OSA [14].

Strengths of our study include the collection of two sputums for evaluation by Xpert and culture and evaluation in a high HIV prevalence setting. Previous buccal OSA evaluations relied on the collection of one sputum tested primarily by Xpert to identify participants with TB [5, 6]. Reduced OSA performance in this Kenyan evaluation could be due to differences in setting, population, and/or study design, as well as the use of a swab type that has since been replaced in subsequent evaluations due to improved performance. The South African study required participants suspected of TB have at least three of the following symptoms: productive cough, unexplained weight loss, chest pain, and hemoptysis to ensure a greater proportion of participants suspected of TB would have microbiologically confirmed TB. This may have identified participants with more advanced TB disease. In contrast, this Kenyan evaluation required only cough for 2 weeks and one additional TB-associated symptom and may reflect a wider more generalizable population presenting to clinic for TB evaluation.

Our study has limitations including small sample size. Given the cross-sectional design, we were unable to determine if OSA-positive participants with initially negative sputum Xpert and/or culture later developed TB. In pediatric OSA evaluation using buccal swabs in South Africa, OSA identified 43% of culture-confirmed TB by induced sputum; but was positive in a substantial proportion of culture-negative children clinically diagnosed with TB, likely identifying additional children with TB missed by sputum samples [7]. As such, some OSA-positive participants in our study with negative Xpert and culture may have actually had culture-negative TB.

While the results of this study using prior techniques did not replicate higher sensitivity reported in the previously published case control study and subsequent cohort evaluation of tongue swabs in South African and Ugandan adults, it does add incrementally to data supporting continued investigation of oral swab sampling and optimization of mucosal site, timing of swab collection, and selection of swab type.

In this study, the intended use of OSA was for TB diagnosis; additional ongoing efforts are evaluating its use as a screening test in the setting household contacts and congregate settings [15]. In an evaluation of tongue swabs used in conjunction with the Xpert platform for mass screening of incarcerated person in Brazil, performance of tongue swabs among Xpert-confirmed participants was 43% and increased to 51% with a second consecutive swab collected the next day, with improved sensitivity among participants with high or medium bacterial load in sputum as measured by Xpert [15]. A trend towards higher sensitivity and stronger signals in subsequent early morning visits has been reported in this study and evaluations in South Africa and China [6, 16]. Previous TB guidelines recommended the collection of an early morning sputum when possible, in addition to “spot” collection to increase the yield of TB diagnostic testing. Early morning collection is thought to improve yield potentially due to diurnal variation in sputum volume or accumulation of sputum overnight, with systematic reviews confirming increased diagnostic performance for microscopy with early morning vs. spot samples [17]. Further evaluation is needed whether the use of early morning self-collected swabs may increase yield among people with TB symptoms who are unable to expectorate sputum on-demand in a clinic setting. The potential clinical role of OSA is as an easy to use sample collection method for people with suspected TB who are unable to produce sputum in settings where more invasive means of sputum collection are unavailable.

## Conclusions

In summary, this study confirms *M*. *tuberculosis* DNA can be detected in the oral cavity of persons with TB, including PLHIV, and provides a promising means of TB detection in populations that may not be able to produce adequate sputum. Tongue swabs and a different swab type showed improved performance in more recent evaluations. Efforts are ongoing to increase process automation including adaptation of OSA with Xpert Ultra [15] that, if successful, could contribute to the promise of non-sputum based diagnostics.

## Supporting information

S1 Table Sputum and OSA sample results by study group and visit. (DOCX)

S2 Table OSA swab positivity by Xpert signal. (DOCX)

S3 Table Mean Cq of OSA swabs by Xpert signal. (DOCX)

S4 Table Mean Cq of OSA swabs by TB status, visit, and HIV status. (DOCX)
