# Supplementary material for: Diagnostic performance of oral swabs for non-sputum based TB diagnosis in a TB/HIV endemic setting
Source: PLoS One. 2022 Jan 13;17(1):e0262123. doi: 10.1371/journal.pone.0262123 (PMC8758000; doi:10.1371/journal.pone.0262123)
Supplement: S1 Table — (DOCX) [file pone.0262123.s001.docx]

| **S1 Table. Sputum and OSA sample results by study group and visit** | | | |
| --- | --- | --- | --- |
|  | **Total**^a^  **N=100** | **TB^b^**  **N=20** | **No TB^c^**  **N=80** |
|  | **n/N (%)** | **n/N (%)** | **n/N (%)** |
| **Smear (any pos)** | 13/100 (13.0) | 13/20 (65.0) | 0 (0) |
| Visit 1^d^ | 11/100 (11.0) | 11/20 (55.0) | -- |
| Visit 2^e^ | 11/98 (11.2) | 11/20 (55.0) | -- |
| **MTB culture (any pos)** | 19/100 (19.0) | 19/20 (95.0) | 0 (0) |
| Visit 1 | 18/100 (18.0) | 18/20 (90.0) | -- |
| Visit 2 | 19/98 (19.4) | 19/20 (95.0) |  |
| **NTM culture (any pos)** | 14/100 (14.0) | 1/20 (5.0) | 13/80 (16.3) |
| Visit 1 | 7/100 (7.0) | 1/20 (5.0) | 6/80 (7.5) |
| Visit 2 | 8/98 (8.2) | 0/20 (0) | 8/78 (10.3) |
| **Contaminated culture** | 6/100 (6.0) | 0/20 (0) | 6/80 (7.5) |
| Visit 1 | 4/100 (4.0) | 0/20 (0) | 4/80 (5.0) |
| Visit 2 | 5/98 (5.1) | 0/20 (0) | 5/78 (6.4) |
| **Xpert MTB/RIF (any pos)** | 17/100 (17.0) | 17/20 (85.0) | 0 (0) |
| Visit 1 | 15/17 (75.0) | 15/20 (75.0) | -- |
| High | 3/15 (20.0) | 3/20 (15.0) | -- |
| Medium | 7/15 (46.7) | 7/20 (35.0) | -- |
| Low | 5/15 (33.3) | 5/20 (25.0) | -- |
| Very low | 0/15 (0) | 0/20 (0) | -- |
| Visit 2 | 16/100 (16.33) | 16/20 (80.0) | -- |
| High | 1/16 (6.3) | 1/20 (5.0) | -- |
| Medium | 9/16 (56.3) | 9/20 (45.0) | -- |
| Low | 3/16 (18.8) | 3/20 (15.0) | -- |
| Very low | 3/16 (18.8) | 3/20 (15.0) | -- |
| **OSA buccal swab (any pos)** | 28/100 (28.0) | 13/20 (65.0) | 15/80 (18.8) |
| Visit 1 | 21/100 (21.0) | 10/20 (50.0) | 11/80 (13.8) |
| Visit 2 | 18/98 (18.4) | 12/20 (60.0)) | 6/78 (7.7) |
| NTM – Non-tuberuclosis mycobacterium  ^a^ Participants suspected of TB with cough >2weeks as well as at least one additional symptom (fever, weight loss, night sweats, or hemoptysis) had 2 visits with sputum collected for smear, Xpert, culture  ^b^ Either sputum culture or Xpert positive for *M. tuberculosis*  ^c^ Negative sputum culture and Xpert for *M. tuberculosis*  ^d^ Enrolment visit  ^e^ Subsequent morning visit | | | |
