# Supplementary material for: Diagnostic performance of oral swabs for non-sputum based TB diagnosis in a TB/HIV endemic setting
Source: PLoS One. 2022 Jan 13;17(1):e0262123. doi: 10.1371/journal.pone.0262123 (PMC8758000; doi:10.1371/journal.pone.0262123)
Supplement: S2 Table — (DOCX) [file pone.0262123.s002.docx]

| **S2 Table. OSA swab positivity by sputum Xpert signal** | | | |
| --- | --- | --- | --- |
|  | **OSA+/Xpert+**  **n/N (%)** | | |
|  | **Total** | **Visit 1** | **Visit 2** |
| **Total** | 21/31 (67.7) | 9/15 (60.0) | 12/16 (75.0) |
| **Xpert signal** |  |  |  |
| Very low | 2/3 (66.7) | -- | 2/3 (66.7) |
| Low | 5/8 (62.5) | 1/5 (20.0) | 3/3 (100) |
| Medium | 11/16 (69.0) | 5/7 (71.4) | 6/9 (66.7) |
| High | 4/4 (100) | 3/3 (100) | 1/1 (100) |
