# Supplementary material for: Diagnostic performance of oral swabs for non-sputum based TB diagnosis in a TB/HIV endemic setting
Source: PLoS One. 2022 Jan 13;17(1):e0262123. doi: 10.1371/journal.pone.0262123 (PMC8758000; doi:10.1371/journal.pone.0262123)
Supplement: S3 Table — (DOCX) [file pone.0262123.s003.docx]

| **S3 Table. Mean Cq of OSA swabs by sputum Xpert signal** | | |
| --- | --- | --- |
| **Xpert signal** | **Visit 1** | **Visit 2** |
| Very low | -- | 34.8 + 2.6 |
| Low | 38.4 + n/a | 34.4 + 1.7 |
| Medium | 34.8 + 3.0 | 32.8 + 4.4 |
| High | 34.6 + 2.5 | 29.6 + n/a |
