# Supplementary material for: Diagnostic performance of oral swabs for non-sputum based TB diagnosis in a TB/HIV endemic setting
Source: PLoS One. 2022 Jan 13;17(1):e0262123. doi: 10.1371/journal.pone.0262123 (PMC8758000; doi:10.1371/journal.pone.0262123)
Supplement: S4 Table — (DOCX) [file pone.0262123.s004.docx]

| **S4 Table. Mean Cq of OSA swabs by TB status, visit, and HIV status** | | | |
| --- | --- | --- | --- |
| **Comparison of mean Cq of OSA swabs by TB status and visit** | | | |
|  | **TB** | **No TB** | **p** |
| Visit 1 | 35.4 + 3.5 | 38.4 + 3.5 | 0.03 |
| Visit 2 | 33.2 + 3.6 | 39.2 + 2.9 | <0.0001 |
| **Comparison of mean Cq of OSA swabs by visit** | | | |
|  | **Visit 1** | **Visit 2** | **p** |
| TB | 35.2 + 2.9 | 33.4 + 3.7 | 0.009 |
| No TB | 40.3 + 4.3 | 38.9 + 2.5 | 0.40 |
| **Comparison of mean Cq of OSA swabs by TB and HIV status** | | | |
| **Visit 1** | **HIV+** | **HIV-** | **p** |
| TB | 33.8 + 2.7 | 36.0 + 2.7 | 0.26 |
| No TB | 37.6 + 3.1 | 40.3 + 4.1 | 0.16 |
| **Visit 2** |  |  |  |
| TB | 30.9 + 2.5 | 34.3 + 3.6 | 0.11 |
| No TB | 39.1 + 2.9 | 39.1 + 3.0 | 0.98 |
